# Uncovering the Invisible: Mono-ADP-ribosylation Moved into the Spotlight

**DOI:** 10.3390/cells10030680

**Published:** 2021-03-19

**Authors:** Ann-Katrin Hopp, Michael O. Hottiger

**Affiliations:** Department of Molecular Mechanisms of Disease (DMMD), University of Zurich, 8057 Zurich, Switzerland; AHopp@cemm.oeaw.ac.at

**Keywords:** NAD, NADH, ADP-ribose, ADP-ribosylation, MARylation, PARylation, ART, PARP, competition, cancer, immunofluorescence, macrodomain, Af1521

## Abstract

Adenosine diphosphate (ADP)-ribosylation is a nicotinamide adenine dinucleotide (NAD^+^)-dependent post-translational modification that is found on proteins as well as on nucleic acids. While ARTD1/PARP1-mediated poly-ADP-ribosylation has extensively been studied in the past 60 years, comparably little is known about the physiological function of mono-ADP-ribosylation and the enzymes involved in its turnover. Promising technological advances have enabled the development of innovative tools to detect NAD^+^ and NAD^+^/NADH (H for hydrogen) ratios as well as ADP-ribosylation. These tools have significantly enhanced our current understanding of how intracellular NAD dynamics contribute to the regulation of ADP-ribosylation as well as to how mono-ADP-ribosylation integrates into various cellular processes. Here, we discuss the recent technological advances, as well as associated new biological findings and concepts.

## 1. ADP-Ribosylation (Introduction)

ADP-ribosylation (ADPR) is a covalent chemical modification conserved throughout all domains of life except budding yeast [1]. The modification was initially identified as a post-translational protein modification, though it has recently been found on nucleic acids as well [2,3,4,5,6]. ADP-ribosylation is catalyzed by ADP-ribosyltransferases (ARTs) and consists of the transfer of ADP-ribose (ADPr) from nicotinamide adenine dinucleotide (NAD^+^) onto the substrate with subsequent release of nicotinamide (NAM) [7,8,9]. NAD^+^ and its reduced form NADH are both important redox equivalents and key cofactors for the electron transport chain, thereby fueling oxidative phosphorylation (OXPHOS) [10,11]. Therefore, the dependence of ADP-ribosylation on NAD^+^ directly links the modification to cell metabolism. ADP-ribosylation comes in two different flavors: while the attachment of a single ADP-ribose molecule is referred to as mono-ADP-ribosylation (MARylation), the gradual attachments of multiple ADP-ribose moieties onto one another results in oligo or poly-ADP-ribosylation (PARylation) [8,12].

**Figure 1:**
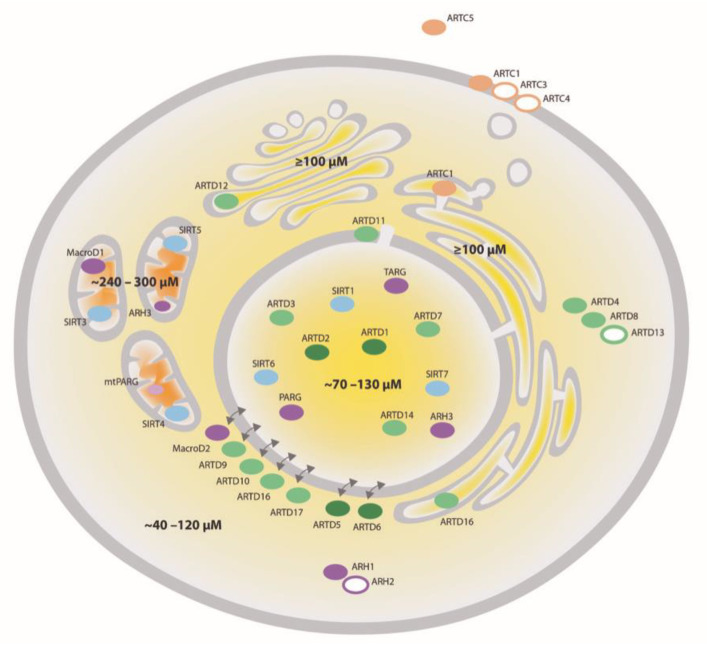
Compartmentalization of NAD^+^-converting enzymes and NAD^+^. NAD^+^-converting enzymes have been identified in different cellular compartments. ARTDs are colored in green, ARTCs—in beige, sirtuins—in blue and ADP-ribosylhydrolases (ARHs)—in purple. Filled circles symbolize active enzymes while open circles indicate enzymes whose activity has not been confirmed so far. The intensity of the color shows the expected intensity of the enzymatic activity. The concentration of NAD^+^ (depicted in different yellow to orange shades) is high in mitochondria (∼300 μM), intermediate in the nucleus and cytosol (∼100 μM) and low (<1 μM) in the extracellular space and can vary considerably depending on the cell type, metabolic condition, stress and redox status. Poly-ADP-ribose glycohydrolase (PARG); Terminal ADP-ribose protein glycohydrolase (TARG).

Mammalian ARTs (i.e., writers) have classically been divided into three groups: (i) clostridium toxin-like ARTs (ARTCs) are mainly described to catalyze extracellular ADP-ribosylation, while (ii) diphtheria toxin-like ARTs (ARTDs or poly-ADP-ribose polymerases (PARPs)) and (iii) sirtuins Sirt 4, 6 and 7 catalyze ADP-ribosylation in different intracellular compartments [12,13,14]. Among those enzymes, only four proteins (ARTD1/PARP1, ARTD2/PARP2 and ARTD5 and 6/tankyrase 1 and 2) have been reported to possess PARylation activity [12]. With the exception of ARTD13/PARP13, whose activity could not yet be detected, all remaining intra- and extracellular ARTs and sirtuins catalyze MARylation [15] (Figure 1).

In addition to these enzymes, recent studies have proposed that other protein families, including NEURL4-like enzymes and certain leucine-rich repeat-containing enzymes, are able to catalyze ADP-ribosylation as well [16,17]. In recent years, protein ADP-ribosylation has emerged as a complex and dynamic post-translational modification that either directly affects the modified proteins or leads to modification-dependent protein complex formation thus serving as a signaling molecule [18]. ADP-ribosylation-dependent signaling often involves so-called readers―proteins that contain specific domains able to bind the mono-ADP-ribose (MAR) and/or the poly-ADP-ribose (PAR) [12,19]. Representative domains such as macrodomains, WWE domains or PAR-binding zinc fingers selectively recognize different forms of ADP-ribose such as MAR, PAR and oligo-ADP-ribosylation.

ADP-ribosylation is believed to be a reversible modification. The removal of ADP-ribosylation is mediated by ADP-ribosylhydrolases (ARHs, e.g., eraser) that either hydrolyze PAR or MAR or both [20,21,22,23,24]. MAR-specific ARHs seem to function in an ADPr amino acid acceptor site-specific manner [25]. The mammalian ARHs can be classified into ADP-ribosyl glycohydrolases and macrodomain-containing hydrolases, including their most prominent member, poly-ADP-ribose glycohydrolase (PARG), and its different isoforms [12]. As for the writers of ADP-ribosylation, the erasers also localize throughout the whole cell, such that together they cover every subcellular compartment (Figure 1). Amongst the ARHs, ARH1 and the inactive ARH2 localize to the cytoplasm, while ARH3 was shown to be nuclear and partially mitochondrial [26]. The macrodomain-containing ARH MacroD1 was shown to be mainly mitochondrial, while MacroD2 is cytoplasmic and TARG localizes to the nucleus [27,28,29]. Depending on the isoform, PARG, the major PAR degrading enzyme, either localizes to the nucleus, the cytoplasm or mitochondria [30]. Interestingly, MAR and PAR are also in vitro substrates for Nudix hydrolases and phosphodiesterases, although in this reaction, the modification is not completely removed, generating phosphoribosyl-modified proteins [31,32].

Although writers, readers and erasers of intracellular mono-ADP-ribosylation have been identified only recently, it is becoming more and more evident that this reversible post-translational modification is involved in a plethora of physiological and pathophysiological processes. More specifically, the modification plays vital roles in regulating cellular stress responses related to the quality control of DNA, RNA and proteins, such as the DNA damage response and the cytoplasmic stress response [1]. In addition, ADP-ribosylation is involved in host–pathogen interactions and several inflammatory signaling pathways [9,33]. In non-stress and non-self-defense pathways, ADP-ribosylation contributes to the regulation of cell cycle progression, gene expression, telomere length and protein stability [34]. Finally, ADP-ribosylation has also been shown to favor pathophysiological conditions such as tumorigenesis and tumor progression, in which it has been described to regulate the unfolded protein response (UPR) [35], the cytoplasmic stress response, miRNA-mediated post-transcriptional gene regulation [36], cancer-related signal transduction pathways or cell migration [37].

An increasing interest in uncovering the precise molecular mechanisms of how protein ADP-ribosylation and particularly MARylation and the responsible ARTs involved in its turnover integrate with the various processes mentioned above has pushed the development and improvement of innovative ADP-ribosylation and NAD^+^ detection and quantification tools within the last five years. In this review, we will discuss the recent technological advances as well as associated biological findings of protein ADP-ribosylation particularly catalyzed by the ART family (i.e., ARTD or ARTC).

## 2. NAD^+^ Synthesis and Its Involvement in Redox Reactions

Since NAD^+^ is the only known ADP-ribose donor, ADP-ribosylation is tightly linked to the availability and subcellular distribution of NAD^+^ pools. In fact, in cell culture, the turnover of ADP-ribosylation was found to directly correlate with overall NAD^+^ levels and NAD^+^ synthesis [38]. NAD^+^ availability depends on two factors: synthesis capacity and the cellular redox state.

### 2.1. NAD^+^ Synthesis and NAD^+^-Synthesizing Enzymes

In cells, NAD^+^ can be synthesized de novo from tryptophan, via the Preiss–Handler pathway from nicotinic acid or from its breakdown products nicotinamide or nicotinamide riboside (NR) via the so called salvage pathway [39,40,41]. The salvage pathway is especially important for the restoration of intracellular NAD^+^ pools following extensive enzymatic consumption, e.g., upon hyperactivation of ARTD1 and subsequent hyper-consumption of NAD^+^. The choice of NAD^+^ synthesis pathways depends on the expression pattern of the respective enzymes involved in either pathway and was shown to be highly cell type- and organ-specific. With the exception of the liver where NAD^+^ levels were shown to predominantly depend on tryptophan, many tissues and most transformed cell culture cell lines synthesize NAD^+^ from NAM and thus rely mostly on the salvage pathway [38]. The salvage pathway depends on the expression level of nicotinamide phosphoribosyltransferase (NAMPT) and the nicotinamide mononucleotide adenylyl transferases (NMNATs), NMNAT1, 2 and 3 [42,43]. The localization of the three NMNATs is described to be enzyme-specific, with NNMAT1 localizing mainly to the nucleus, NMNAT2—to the cytoplasm as well as to the Golgi apparatus and NMNAT3—to the mitochondria [44,45,46]. While NAMPT is mostly found in the nucleus and the cytoplasm, a small fraction is believed to be mitochondrial, thus enabling all three compartments to fully resynthesize NAD^+^ from NAM. Given that many small metabolites are believed to freely diffuse between the nucleus and the cytoplasm, nuclear and cytoplasmic NAD^+^ pools are thought to be interconnected. In line with that, in certain cell culture settings, NMNAT1 can compensate for the lack of NMNAT2 and vice versa. In strong contrast to those in vitro experiments, knockout of either NMNAT1 or NMNAT2 in mice is lethal [47,48]. Hence, although NAD^+^ is thought to freely shuttle between the nucleus and the cytoplasm in vivo, neither enzyme seems to be able to functionally compensate for the loss of the respective other one. Comparably, mutations in either the NMNAT1- or the NMNAT2-encoding gene in humans were shown to result in the early development of severe disorders [49,50], again suggesting that the regulation of these proteins is compartmentalized and non-redundant in vivo. Recent studies have now challenged the well-established role of NMNAT-catalyzed NAD^+^ production and might provide explanations for the above-described discrepancies between in vitro and in vivo studies. Those reports propose that NMNATs additionally exert important chaperone functions that are critical for neuronal health [51,52,53]. To which extent this potential chaperone function has to be taken into consideration for experiments in cell culture has to further be assessed by including NMNAT mutants that can discriminate between the two functions.

### 2.2. NAD^+^ Oxidation and Reduction

In cells, both NAD^+^ and NADH exist in either a free or a protein-bound form. Redox reactions that convert NAD^+^ to NADH or vice versa are catalyzed by over 700 oxidoreductive enzymes. Those enzymes play critical roles in multiple biological processes, including energy metabolism, mitochondrial function, biosynthesis, gene expression, calcium homeostasis, cell death, aging and carcinogenesis [11,54,55,56,57,58,59,60]. The NAD^+^/NADH ratio was estimated to be approximately 700–1000 in the nucleus/cytosol and 7–8 in mitochondria [59,61,62]. Changes in intracellular NAD^+^/NADH ratios are thus closely linked to the overall fitness of a cell (particularly, the mitochondria) and do not only affect cell metabolism, but might also affect ADP-ribosylation [59,60,63,64,65,66,67,68]. The nuclear and cytoplasmic NAD^+^/NADH ratios are predominantly controlled by three major processes: glycolysis, lactate dehydrogenase activity and mitochondrial NADH shuttles [69], which allow the translocation of electrons produced during glycolysis across the semipermeable inner membrane of mitochondria. Beside the malate aspartate shuttle, nuclear/cytosolic and mitochondrial NAD^+^ and NADH pools were long believed to be strictly spatially separated. As clear evidences for the existence of a mitochondrial NAD^+^ transporter were missing, nuclear/cytoplasmic and mitochondrial NAD^+^ pools were considered to not be exchangeable [70]. The recent identification of the NAD^+^ transporter SLC25A51 that localizes to the mitochondrial inner membrane has, however, demonstrated that a direct NAD^+^ exchange between mitochondria and the cytoplasm is possible and potentially physiologically relevant for cells [71,72,73].

## 3. NAD^+^ Quantification

Given the vital role of NAD in many cellular processes, it is not surprising that NAD^+^ levels were shown to be decreased in aged individuals and supplementation with NAD^+^ precursors was proven to ameliorate age-related disorders, including neurodegenerative diseases [74,75,76]. In line with that, the function of NAD^+^-dependent enzymes, such as sirtuins and ARTs, was shown to change upon aging [77]. Therefore, to be able to study NAD^+^ dynamics in the precise (patho-) physiological contexts, effort has been put into developing tools to facilitate NAD^+^ quantification and the assessment of NAD^+^/NADH ratios in various cellular systems and different subcellular compartments. The developed methods and tools can roughly be divided into three different categories: (i) chemical luminescence-based assays, (ii) liquid chromatography (LC)-based and LC–mass spectrometry (LC/MS)-based targeted metabolomics and (iii) genetically encoded fluorescent sensors. All three approaches are summarized in Table 1 and discussed in more detail in the following subsection. As the various methods described in the following section differ in their experimental setup and detection strategy, the scientific question should guide the choice of detection tool.

### 3.1. Chemical Luminescence-Based Assays

For many years, investigators have been indirectly measuring the ratios of NAD^+^/NADH by using luminescence-based chemical methods that deduct the NAD^+^/NADH ratio indirectly from the activity of redox couples such as lactate and pyruvate [62]. Remarkably, in contrast to mitochondria, nuclear/cytosolic NADH levels are extremely low (NAD^+^/NADH ratio of 700–1000 in the nucleus/cytosol and of 7–8 in mitochondria) [62,86], with the majority being protein-bound and therefore rather static [59]. However, these methods require the use of cell extracts and thus only allow the determination of total amounts of NAD^+^/NADH without providing information with respect to their subcellular location. For the same reason, these methods cannot be used to study NAD^+^ dynamics in intact individual cells. Furthermore, such indirect chemical methods cannot distinguish between protein-bound and free species of NAD^+^ or NADH. Since only free NAD^+^ can be used for ADP-ribosylation, measurement of total NAD^+^ and NADH does not allow making statements with respect to the ADP-ribosylation capacity of a cell/organelle. The need to measure NAD^+^ and NADH independently from one another represents a major reason for the development of individual sensors able to measure changes in free NAD^+^/NADH ratios [87], Nicotinamide adenine dinucleotide phosphate (NADP^+^) [88], Nicotinamide adenine dinucleotide phosphate hydrogen (NADPH) [89], NADH [90,91], as well as NAD^+^ [83,84]. This new generation of sensors is particularly interesting for the study of ADP-ribosylation (see below).

### 3.2. Liquid Chromatography (LC) and LC–Mass Spectrometry-Based NAD Measurements

Several research groups have established LC-based [78] as well as targeted LC/MS-based methods comprising the use of isotope-labeled NAD^+^ or NAD derivatives to quantify the intracellular NAD^+^ level and NAD^+^/NADH ratios [79,80,81]. Comparable to the chemical assays, those MS-based technologies also require the generation of cell lysates and thus do not allow assessing NAD^+^ dynamics over time and following different treatments. In addition, unless organelle purification strategies are included in the sample preparation workflow, the distinction between NAD^+^ levels in different subcellular compartments is not possible. Since NAD^+^ and its derivatives rapidly transition between one another and often are similar in terms of molecular weight, a precise distinction between different NAD^+^ derivatives remains challenging. A recent publication in which isotope-labeled NAM, nicotinic acid or nicotinamide riboside was given to cells or mice enabled for the first time to measure NAD^+^ synthesis and breakdown fluxes in various organs and cell types [38]. Although this approach can infer NAD turnover in vivo based on its labeling kinetics from isotopic NAM, it did not dissect the consuming enzymes involved.

### 3.3. Genetically Encoded Fluorescent Sensors

Since only free NAD^+^ is relevant for ADP-ribosylation, the first genetically encoded NAD^+^ sensor consisted of the catalytic domain of human ARTD1 that was targeted to various cellular organelles, for instance, mitochondria [82]. The method is based on a qualitative rather than quantitative detection of polymers (i.e., PAR-assisted protein localization assay, PARAPLAY). Immunochemical detection of PAR chain formation in mitochondria, peroxisomes and, surprisingly, in the endoplasmic reticulum (ER) and the Golgi apparatus demonstrated the existence of multiple subcellular NAD^+^ pools. However, as ARTD1 is a potent NAD^+^ consumer, the use of this sensor does affect NAD^+^ concentrations in the targeted organelles and, therefore, might interfere with the energetic state of the cell.

The first genetically encoded biosensor that does only bind but not consume NAD^+^ consists of a circularly permuted Venus fluorescent protein (cpVenus) and the bipartite NAD^+^-binding domain derived from the bacterial DNA ligase [83]. Targeting this cpVenus-based NAD^+^ biosensor to various subcellular compartments allowed estimating the respective NAD^+^ level to be 92–122 μM in the cytoplasm, 87–136 μM in the nucleus and 191–275 μM in the mitochondria [83].

Almost in parallel, a second genetically encoded fluorescence resonance energy transfer (FRET)-based semi-synthetic biosensor was developed based on the “Snifit” concept [84,85]. Different variants of the sensor enable the quantification of free NAD^+^ and ratios of reduced to oxidized nicotinamide adenine dinucleotide phosphate (NADP) in living cells [84]. As for the other sensors, the constructs can be targeted to different subcellular localizations, thereby allowing measuring the respective NAD^+^ concentrations. The FRET-based sensors possess a large dynamic range, can be excited at long wavelengths and are pH-insensitive. Using this NAD^+^ biosensor, free intracellular NAD^+^ levels in U2OS cells were found to be around 70–120 µM [84]. Free cytosolic NAD^+^ of different tested cell lines were found to be relatively similar, ranging from 40 to 70 µM (Figure 1). With respect to NADPH/NADP^+^, free NADPH/NADP^+^ was discovered to be maintained at a high ratio inside cells while the reduction potential of mitochondria was significantly higher than that of the cytosol and the nucleus. The higher ratio of NADPH/NADP^+^ in mitochondria could, at least partially, be due to the higher pH in that organelle, pushing mitochondrial NAD(P) transhydrogenases and dehydrogenases towards the formation of NADPH [92]. Free cytosolic NADPH/NADP^+^ ratios in the different cell lines varied up to fourfold, ranging from 20 to 80 [84]. Overall, these values provide a foundation for future efforts to map the metabolic state of different cell types and organelles. Remarkably, despite the very different designs of the two NAD^+^ biosensors discussed above, there was a strong congruence in the measured concentration of free NAD^+^ (40–70 μM NAD^+^) in the cytoplasm of various cell lines (e.g., HeLa, Human embryonic kidney (HEK)293T, U2OS) [83,84] (Figure 1).

## 4. Regulation of Cellular MARylation

The establishment of protein MARylation can be regulated on various levels. First, expression and cellular distribution of enzymes largely contribute to controlling the abundance of the enzymes, their localization and their target spectrum. Second, the ADP-ribosylation ability of every ART is naturally linked to the availability of NAD^+^ and its *Km* for NAD^+^ that varies from enzyme to enzyme and ranges from only few µM to several hundred µM [93,94,95,96]. Finally, comparable to other enzymes, mono-ARTs can be regulated by additional signaling events and binding to cofactors ([35,97,98] and reviewed in [99,100]). The regulation of MARylation by ART expression and NAD^+^ affinity or NAD^+^ competition is discussed in the following sections.

### 4.1. Transcriptional Regulation of the ARTs Catalyzing MARylation

Strikingly, a large number of mono-ARTs were found to be upregulated in response to specific stimuli. Lipopolysaccharides (LPS)/ Interferon (IFN)γ, for instance, increased the mRNA expression of ARTD8/PARP14 and subsequently induced IRF3 signaling-regulated primary response genes, including ARTD14/PARP7, ARTD16/PARP8, ARTD10/PARP10, ARTD11/PARP11, ARTD12/PARP12 and ARTD13/PARP13 in murine bone marrow-derived macrophages [101] (see also Chapter 7). Comparably, mouse hepatitis virus (MHV) infection or stimulation of cells with interferon strongly induced expression of the mono-ARTs ARTD14, ARTD9/PARP9, ARTD10, ARTD11, ARTD12, ARTD13 and ARTD8 [102,103,104,105]. Ferrets infected with severe acute respiratory syndrome coronavirus (SARS-CoV)-2 showed elevated expression of intracellular mono-ARTs as well, suggesting that this phenomenon is also relevant in vivo [106]. Specifically, ARTD4/PARP4, ARTD5(6)/PARP5, ARTD14, ARTD9, ARTD11, ARTD13, ARTD8 and ARTD7/PARP15 were all significantly induced [107]. Other reports linked ARTD10 and ARTD12 to NF-κB signaling [108,109]. Consistent with the role of other post translational modifications (PTMs) in immunity, the above-described findings revile intracellular mono-ARTs to play important roles in the restriction of viral replication and to regulate viral infectivity and pathogenesis [103,104,110,111,112,113,114,115,116,117,118].

Little is known about the expression regulation of the remaining intra- and extracellular mono-ARTs (e.g., ARTD3/PARP3, ARTD17/PARP6, ARTD15/PARP16 or ARTC1, respectively) [98,119,120]. ARTD3 expression seemed to be regulated by TGF β and thus to be important in epithelial-to-mesenchymal transition (EMT) [121,122]. ARTD15 seems to be constitutively expressed and involved in the ER stress responses and, potentially, in nuclear transport [35,123].

While the expression of ARTC1 during muscle differentiation is regulated by the myogenic transcription factors MEF-2 and myogenin [124], stimulation of alveolar epithelial cells with immunogens (lipoteichoic acid (LTA), flagellin or LPS) also increased ARTC1 expression levels [125]. The regulation of ARTC1 in the context of stem cell-regenerative responses was further recently described to be dependent on the osteosarcoma oncogene (*Fos*) [126].

### 4.2. NAD^+^ Affinity-Driven Regulation of Intracellular MARylation

Considering that intracellular NAD^+^ levels lie between 40 to 120 µM in the nucleus and the cytoplasm and up to almost 250 µM in mitochondria [83,84], ARTs with a *Km* for NAD^+^ below this concentration are unlikely to be regulated by cellular NAD^+^ levels and are potentially constitutively active if their activity were to depend solely on NAD^+^. In line with that, the auto-modification activity of different mono-ARTs can be detected upon overexpression in cells [127], thus confirming that the existing cellular free NAD^+^ levels are enough to sustain even high amounts of intracellular MARylation. Of note, as overexpression of a given protein often largely exceeds the endogenous levels, a potential regulation by a cofactor cannot be excluded for the respective enzymes due to stochastic imbalances. In contrast, those ARTs whose *Km* for NAD^+^ is higher than the concentration of free NAD^+^ in a given subcellular compartment (e.g., ARTD12 and ARTD15 with 299 μM and 582 μM, respectively [93,95]) are likely inactive under homeostatic conditions and require additional regulation (see below). The lack of some mono-ART-associated MARylation activity might, however, also be the result of a very fast turnover of this modification due to the presence of a MARylation-specific ARH or rapid degradation of the modified target protein.

Recent studies were able to provide evidence for the existence of NAD^+^ in the extracellular milieu (extracellular NAD^+^, eNAD^+^) [128]. Despite the presence of dimerized extracellular NAMPT (eNAMPT), which can generate nicotinamide mononucleotide (NMN) [129], the enzymes necessary to catalyze the final step of extracellular NAD^+^ synthesis are currently unknown. While the basal eNAD^+^ was quantified to be around 0.1 μM [70,128], eNAD^+^ as well as other nucleotides can be released by controlled mechanisms during hypoxia and inflammation [130,131]. However, once released, eNAD^+^ is rapidly degraded by ectoenzymes such as the NADases CD38 and CD157 [132,133]. Thus, although the *Km* of membrane-bound ARTC1 for NAD^+^ was described to be much lower than the one of the intracellular ARTs, between 5–12 μM [134,135], the enzyme is most likely still inactive under unperturbed conditions. Under extremely stressful conditions, including lytic processes, such as tissue damage and necrosis, high amounts of intracellular NAD^+^ can be released into the extracellular space, thus enabling the activation of ectopic ARTC enzymes [130,131]. Recent reports indeed suggested that the ectopic ARTC1 ADP-ribosylates TRIM72 upon plasma membrane rupture and subsequent release of intracellular NAD^+^, thereby playing a critical role in plasma membrane repair [136]. In addition to this stress-induced activation mode, ARTC1 might also be able to modify certain target proteins already in the ER or the Golgi apparatus before the enzyme and its target are transported to the plasma membrane, since the NAD^+^ levels are expected to be higher in these compartments [137]. The first evidence for this possibility has recently been reported [138].

### 4.3. NAD^+^ Competition-Based Regulation of Intracellular MARylation

In addition, coexistence of several NAD^+^-consuming enzymes in the same compartment provides the third, competition-based regulation layer. Based on the low *Km* of nuclear ARTD1 for NAD^+^ (described to vary between 20 μM and 50 μM [94,139,140]), it seems to be constitutively active and the main NAD^+^ consumer in homeostatic conditions [141,142]. Consequently, ARTD1 forces the cell to continuously synthesize NAD^+^ de novo or via the salvage pathways to maintain cellular viability and allowing other ARTs to be active [143]. Due to this resynthesis of intracellular NAD^+^, MARylation mediated by other mono-ARTs with high affinity for NAD^+^ is still possible despite continuous ARTD1-mediated NAD^+^ consumption.

Upon genotoxic stress, binding of ARTD1 to DNA breaks is reported to stimulate its activity by more than 500-fold [139]. The resulting strong reduction in free nuclear and cytoplasmic NAD^+^ levels might coregulate the activity of other ARTs or other NAD^+^-dependent enzymes (e.g., Sirt1 *Km* for NAD^+^ ∼ 150–280 μM [144,145]). Since, based on their elevated *Km*, most of those enzymes require elevated NAD^+^ levels to enhance their activity, they are suitable to act as metabolic NAD^+^ sensors [146,147]. Reduced NAD^+^ levels following ARTD1-mediated hyper-consumption might even affect ARTD1 itself. This possibility is in agreement with recent reports, suggesting that even upon DNA damage, ARTD1 predominantly catalyzes protein MARylation rather than PARylation [148]. Interestingly, overexpression of ARTD10 that led to enhanced mono-ADP-ribosylation was sufficient to repress cellular NAD^+^ levels and, consequently, the MARylating activities of ARTD14, ARTD10, ARTD12 and ARTD8 [107]. The effect could be reversed by stimulating NAD^+^ synthesis via pharmacologically activating NAMPT [107,149].

Opposite to the above-described scenario, intracellular MARylation activity could also be potentially influenced by transiently increasing the free NAD^+^ level, for example, via inhibition or genetic ablation of ARTD1. Indeed, NAD^+^ level are up to two times higher in ARTD1 knockout mice [150]. Along that line, the activity of Sirt1 was reported to inversely correlate with the activity of ARTD1 [151]. Thus, inhibition of ARTD1 could indeed result in an increased ADP-ribosylation potential mediated by other ARTs. Alternatively, a local increase of NAD^+^ could be achieved by synthesis. While NMNAT1 has been described to associate with ARTD1, thereby most likely providing elevated local NAD^+^ concentration in close proximity of the enzyme [152,153], a similar mechanism has not yet been described for mono-ARTs.

### 4.4. Regulation of Intracellular MARylation by Intracellular NAD^+^ Redistribution

In addition to the above-described direct NAD^+^-dependent and NAD^+^ competition-dependent regulatory mechanisms, it is possible that, following specific types of stimulation, cells reshuffle different intracellular NAD^+^ pools [154]. That way, NAD^+^ derived from compartments with high basal concentrations can be used to support NAD^+^-dependent processes in other compartments with lower basal concentrations and lower synthesis rate. In fact, our group recently observed that following stimulation with H_2_O_2_, mitochondrial NAD^+^ is relocated to the nucleus in order to sustain the extensive PARylation that is induced following activation of ARTD1 [155]. The same phenomenon was not solely specific to H_2_O_2_, but also happened in response to other treatments that result in extensive activation of ARTD1. Interestingly, forcing cells to increase their mitochondrial ADP-ribosylation dampened H_2_O_2_-induced nuclear PARylation, suggesting that subcellular NAD^+^ redistribution can be used by cells to modulate their ADP-ribosylation potential in different compartments. Given that mitochondria have been shown to locally interact with other subcellular compartments, such as the ER, it is interesting to speculate that similar events happen between mitochondria and other organelles, too. Our data suggest that mitochondrial ADP-ribosylation is a dynamic process and that NAD^+^ shuffling can be used to orchestrate the activity of ARTs localized to different subcellular compartments.

## 5. Detection of Mono-ADP-Ribosylation

The two main challenges for the development of ADP-ribosylation detection tools can be attributed to the heterogenic nature of ADP-ribosylation (MARylation vs. PARylation) and similarity of this molecule to several other abundant cellular biological (macro-)molecules, including adenine nucleotides and nucleic acids [156,157]. Many antibodies against poly-ADP-ribose can thus recognize RNA and DNA and vice versa [158,159,160]. The heterogeneity can be attributed to the fact that ADP-ribosylation is catalyzed by at least 25 distinct enzymes with cell type-specific expression patterns and different cellular localizations and targets, as well as amino acid preferences. The currently available ADP-ribosylation tools can roughly be divided into three different categories, which are summarized in Table 2: (i) anti-ADP-ribose or PAR-binding antibodies, (ii) ADP-ribose-binding domains and (iii) chemical labeling of ADP-ribose. All three approaches and the most recent advances made in each category are discussed in the following sections.

### 5.1. Antibodies against MAR and/or PAR

While the first PAR-detecting antibody was developed over 40 years ago [161], the generation of MARylation-specific antibodies has proven to be difficult. One reason for this is the challenge to synthesize MARylated immunogens to obtain an antibody capable of recognizing MAR that is not cross-reacting with either PAR or adenosine-derived small metabolites and PTMs, such as adenylation. Despite these difficulties, the recent synthesis of peptides with ADP-ribose-like modifications presented the first milestone for the development of pan-ADP-ribose- and MARylation-specific antibodies [172,173]. Those and similar peptides and proteins carrying different ADP-ribose-like units indeed enabled the successful generation of several polyclonal ADP-ribose-recognizing antibodies [127,155]. A comparison between some of these antibodies and the macrodomain Af1521 commonly used for MS analysis, however, revealed that for those analysis, the macrodomain still outperforms the antibodies [174]. Nonetheless, while the new antibodies recognize MAR and PAR and therefore are referred to as pan-ADP-ribose antibodies, they could already be used to study organelle and mono-ART-specific ADP-ribosylation dynamics by Western blotting (WB) and Immunofluorescence (IF) in different contexts [127,175]. A recent study finally reported the generation of several MARylation-specific antibodies that did not cross-react with PAR [148]. The peptides used to generate these antibodies were ADP-ribosylated in vitro, and enzyme promiscuity was prevented by chemically protecting alternative ADPr amino acid acceptor sites. While some of those antibodies generally detect MAR, other were peptide- and ADPr amino acid acceptor side-specific, thus allowing studying the modification in a context-dependent manner. As this study focused on Ser-linked MARylation, it would be interesting to further investigate whether this approach would also be successful for the design of antibodies specific to other ADPr amino acid acceptor sites, including the amino acids Arg and Cys.

### 5.2. ADP-Ribose-Binding Domains

In addition to antibodies, naturally occurring ADP-ribose-binding domains, such as macrodomains or the WWE domain, have been explored with respect to their ability to detect ADP-ribosylation and potentially distinguish between MARylation and PARylation [98,162,163,165]. Among the so far tested ADP-ribose-binding domains, the WWE domain binds iso-ADP-ribose and therefore solely recognizes PAR, while the bacterial macrodomain Af1521 recognizes both MAR and PAR to a similar extent [162]. The 2nd and 3rd macrodomains of ARTD8 efficiently bind MARylated proteins and peptides [162]. Especially when several of the respective macrodomains are combined, the resulting multi-modular binder shows a strong preference of MAR over PAR [98,162,165]. In addition to their preference for different ADP-ribose species (e.g., MAR vs. PAR), the different domains might also partially rely on the context of the modified protein and/or on the ADPr amino acid acceptor side for binding. The identification of different ADPr amino acid acceptor sites with wildtype Af1521, however, rather excludes the later possibility, at least for this macrodomain [176]. Fused to the Fc (fragment crystallizable) region of an antibody, ADPr-binding domains can be used as an efficient tool to detect different forms of ADP-ribosylation by WB or IF [98,162,165]. Moreover, ADPr binders can be used as bait in order to pull down and enrich ADP-ribosylated peptides. In fact, the macrodomain Af1521 has been used for the establishment of the ADPr/chromatin capture method named ADPr chromatin affinity precipitation (ADPr-ChAP) and is commonly used to enrich ADP-ribosylated proteins prior to MS analysis [163,177,178]. Those MS-based approaches indeed resulted in the identification of numerous potentially ADP-ribosylated proteins and allowed the investigation of ART- and stimulus-specific ADP-ribosylomes [20,176,179].

Very recently, random mutagenesis of the macrodomain Af1521 was used to develop an engineered Af1521 (eAf1521) with 1000-fold increased affinity towards ADP-ribose compared to wildtype Af1521 [164]. Its use for the proteomic ADP-ribosylome MS workflow increased the ADP-ribosylated protein identification rates and yielded greater ADP-ribosylome coverage. Furthermore, generation of an eAf1521 Fc fusion protein resulted in the generation of a new antibody-like detection tool to analyze MARylation. Using this eAf1521 Fc fusion protein for immunoblotting and immunofluorescence confirmed the improved detection of cellular ADP-ribosylation that was observed via MS. However, as Af1521 recognizes both MARylation and PARylation almost to the same extent, it would be of great interest to establish the usage of other macrodomains and ADPr binders to distinguish between MAR and PAR. Alternatively, in an approach similar to the one taken by Nowak and colleagues to improve the general binding of Af1521, ADPr binders could be further evolved by successive rounds of randomized mutagenesis followed by phage display-based affinity purification to specifically bind either MAR or PAR [164].

### 5.3. Chemical Labeling of NAD^+^ or ADP-Ribose

As an alternative approach to the development of new ADP-ribosylation detection tools, some studies have investigated in chemically modifying ADP-ribosylation, such that it can be easily detected with the existing tools [166,167,168,169,180]. One way to label ADP-ribosylation is to replace the naturally occurring NAD^+^ with NAD^+^ carrying a desired chemical group suitable for subsequent visualization (e.g., biotin) within its ADPr-containing part. However, as NAD^+^ is not cell-permeable, this approach is only suitable for in vitro modifications and does not allow detecting ADP-ribosylation within cells. Depending on the size and properties of the chemical group, it is further possible that the affinity of ARTs towards the labeled NAD^+^ is reduced as compared to its natural form. In addition, labeling might interfere with ADPr chain formation or branching, thus altering the nature of the modification. To avoid potential alteration of endogenous ADP-ribosylation and to allow for the detection of endogenous ADP-ribosylation within cells, the recently developed enzymatic labeling of terminal ADPr (ELTA) approach aimed at labeling already synthesized free or protein-bound ADPr [166]. Several other approaches also aiming at detecting intracellularly catalyzed ADP-ribosylation exploited the potential of click-it chemistry for further visualization of the modification. Those approaches comprise supplementation of cells with either N^6^-propargyl adenosine which can readily be taken up, metabolized and used for ADP-ribosylation or a clickable aminooxy alkyne probe that can directly recognize ADP-ribosylation [168,169,170]. To further facilitate the incorporation of both modified metabolites into ADP-ribosylation, ARTs were genetically modified to favor NAD^+^ analogues over natural NAD^+^ [167]. In addition to these approaches that involve click-it chemistry, another study investigated in the synthesis of an NR analog that can be taken up by cells and is locally converted to 3′-azido_NAD^+^ by the nicotinamide riboside kinase 1 and NMNAT1 [171]. The authors describe 3′-azido_NAD^+^ to exhibit a high activity and specificity to ARTD1 and ATD2-catalyzed protein ADP-ribosylation and the resulting PAR chains to show a certain degree of resistance towards PARG-mediated degradation. Some of those approaches involve complete cell lysis and thus are neither suitable to monitor dynamic ADP-ribosylation changes nor do they identify ADP-ribosylation in specific subcellular compartments. While all these approaches can be used to detect and enrich ADP-ribosylated proteins, they do not allow a clear distinction between MAR and PAR. Moreover, a direct comparison of the chemical labeling method with the MS-based protocols revealed a little overlap (<10%) [181].

## 6. Recent Advances and New Emerging Biological Concepts Regarding MARylation in the ER and Mitochondria

The advances made in the development of NAD^+^ and ADP-ribose-detecting tools have, within a very short time, pushed the investigation of ADP-ribosylation in pathways and organelles that in the past were proven impossible or, to a large extent, very difficult. Targeting the catalytic domain of human ARTD1 to different cellular organelles, for instance, revealed that subcellular NAD^+^ pools in mitochondria, peroxisomes, the ER and the Golgi apparatus allowed formation of PAR [82]. In line with that, a recently developed macrodomain-based MAR and PAR detection tool revealed a punctual cytoplasmic ADP-ribosylation signal in unperturbed cells [164]. As the same signal was not detected with commercially available anti-PAR antibodies, it most probably stemmed from MAR. Together, both studies suggest the existence of extensive ADP-ribosylation in subcellular compartments other than the nucleus and the cytoplasm.

### 6.1. ADP-Ribosylation in the ER

Optimized MS-based identification of ADP-ribosylated peptides in HeLa cells subjected to H_2_O_2_ treatment resulted in the identification of > 11,000 unique ADP-ribosylated peptides mapping to >7000 ADP-ribosylation sites [176]. Functional enrichment analysis suggested a subcellular localization-based ADPr amino acid acceptor site distribution, where arginine ADP-ribosylation seems to dominate in the ER. However, whether these modifications were catalyzed by mono- or poly-ARTs remains to be defined. Interestingly, the MAR-catalyzing ARTC family members specifically modify their target proteins at arginine residues. ARTCs are typical glycosylphosphatidylinositol (GPI)-anchored ectoenzymes facing the extracellular space or being secreted [182,183]. Nevertheless, protein maturation of these proteins takes place in the ER and ARTCs might thus be important regulators of the MARylation in this compartment as well [184]. This is in line with previous reports suggesting that the ER’s luminal chaperone GRP78/BiP (glucose-regulated protein of 78 kDa/immunoglobulin heavy-chain-binding protein) is a cellular target of human ARTC1 [138]. Moreover, in cells overexpressing ARTC1, ADP-ribosylation staining using macrodomain Af1521 colocalized with the ER-residing GRP78/BiP, thus providing strong evidence that this modification occurs in the ER [138].

### 6.2. ADP-Ribosylation in Mitochondria

Given the high NAD^+^ concentrations as opposed to other subcellular compartments, mitochondria are almost predisposed to utilize ADP-ribosylation for signaling processes. The existence of ADP-ribosylation in mitochondria was first proposed over thirty years ago, when macromolecular NAD^+^-derived aggregates that were believed to be enzymatically catalyzed were identified in lysates of rat liver [185]. Follow-up studies localized this mitochondrial ADP-ribosylation to the matrix as well as to the intermembrane space and also suggested the existence of mitochondrial ADP-ribosylation writers and erasers [186]. Despite those promising first results, the identification of the respective enzymes as well as of potential ADP-ribosylated target proteins has remained challenging. Interestingly, targeting of recombinant ARTD1 to mitochondria also resulted in the formation of PAR chains in the matrix [82]. Additional targeting of recombinant ARH3 to the mitochondrial matrix could reverse the artificially induced PAR, demonstrating that the conditions present in mitochondria support the full life cycle of the modification [187,188]. Furthermore, permeabilization of cells and subsequent incubation with 3′-azido_NAD^+^ allowed detecting mitochondrial-localized PAR chains by confocal microscopy [171]. However, a recent analysis using ARTD1 knockout cells revealed that the observed mitochondrial ADP-ribosylation is most likely not dependent on ARTD1 [155]. To date, besides ARTD1, SIRT4, ARH3, PARG and MacroD1 are also proposed to be involved in the regulation of ADP-ribosylation in mitochondria by catalyzing either its synthesis or removal [28,29,187,188,189,190,191,192,193]. In addition to the potential contribution of those four enzymes (reviewed in detail elsewhere [39]), a recent study proposed NEURL4 as a new ADP-ribosylation catalyzing enzyme within mitochondria [16,17].

While the enzymatic regulation of mitochondrial ADP-ribosylation remains to be fully understood, exploiting the new generation of ADP-ribosylation-specific antibodies, we recently characterized for the first time mitochondrial ADP-ribosylation in different cellular systems by IF and WB and described its dynamics following metabolic challenges, such as respiratory chain inhibition [155]. We further described an NAD^+^-mediated crosstalk between mitochondrial and nuclear ADP-ribosylation and highlighted the importance of mitochondrial NAD^+^ levels for nuclear ARTD1-mediated processes. The dependence of mitochondrial and nuclear ADP-ribosylation on intracellular NAD^+^ shuttling shows how cells can utilize metabolite distribution to govern physiological processes.

## 7. MARylation in Viral Infections

Several mono-ARTs are reported to be induced by IFN and known to have antiviral properties ([194] and Chapter 4.1), suggesting that ADP-ribosylation plays a critical role in host defense responses, particularly during viral infections. Interestingly, several viruses, including Togaviridae, Hepeviridae and CoVs, encode macrodomain-containing hydrolases that potentially counteract ADP-ribosylation-mediated host–pathogen immune responses, thereby showcasing the century-long coevolution between viruses and their host cells [195,196,197]. In the case of CoVs, knockdown of two abundantly expressed ARTs, ARTD12 and ARTD8, led to increased replication of a hydrolase-deficient mutant, but not the wildtype virus. ARTD8 was further also shown to be important for the induction of IFN in mouse and human cells following viral infection, pointing towards a critical role for this PARP in the regulation of innate immunity [102]. Together, the results of this and other studies demonstrate that viral macrodomains are indeed able to counter ADP-ribosylation-mediated IFN-driven antiviral responses [102,110]. To date, many structures of viral macrodomains have been resolved [194]. The globular macrodomain contains a conserved cleft that has been shown to bind ADP-ribose [27,198,199,200]. Some of the most conserved residues between individual macrodomains are located at the surface near the ADP-ribose-binding cleft, and mutations of these residues are suggested to affect the virulence of the virus [194].

Stress granule formation and disassembly are tightly regulated during viral infection, often reflecting the cellular translation status [201,202,203]. Different ARTs, as well as PARG and specific ADP-ribosylated proteins were found to localize to these condensates [36,204], pointing towards a potential role of ADP-ribosylation in stress granule formation. Indeed, the ratio between ARTs and ARHs was found to induce and repress stress granule formation if shifted to one or the other side. Accordingly, the activity of a viral macrodomain containing ARHs was found to reverse cellular ADP-ribosylation [196] and disrupt stress granule formation [205]. Since viral microdomains are distinct from their human counterparts, this might open avenues for the development of a new class of antiviral therapeutics [206].

## 8. MARylation in Cancer

Since the identification of the synthetic lethality interaction between the poly-ART ARTD1 and BRCA1/2, ADP-ribosylation has become subject of numerous studies aiming at dissecting its function in cancer. To date, PARP inhibitors are FDA-approved therapeutics for the treatment of breast and ovarian cancer [207,208,209] and are considered in clinical studies for the treatment of other frequently diagnosed tumor types, including brain, colon, lung and prostate cancer [210,211,212,213,214,215,216,217]. While most studies investigating the role of ADP-ribosylation in cancer focused on PARylation, recent reports also suggest a relevant role of MARylation and mono-ARTs.

### 8.1. A Potential Role of Mono-ARTs in Cancer Progression and Severity

Several recent studies strongly suggest an involvement of mono-ARTs in the development and progression of cancer. ARTD4, as well as ARTD4-associated RNAs, have, for example, been directly linked to multidrug resistance in cancer [218,219]. Additionally, ARTD10 was shown to promote cell proliferation and tumorigenesis through multiple mechanisms, including the regulation of ß-catenin and relieving replication as well as oxidative stress [220,221]. Furthermore, the enzymatic activity of ARTD10 dampened the activation of NF-κB and downstream target genes in response to interleukin-1β and TNFα, thereby potentially regulating the cancer-directed host immune responses [108]. Using chemical genetics, a recent study proposed ARTD14 to favor tumor progression in ovarian cancer [222]. Exploiting the above-described clickable NAD^+^ analogs and a respective analog-specific ARTD14 mutant, the study identified α-tubulin as a MARylation target of ARTD14. ADP-ribosylation of α-tubulin destabilized microtubule formation, potentially promoting ovarian cancer cell growth and motility. Along a similar line, ARTD14 was identified as a safeguard of pluripotency in embryonic stem cells by protecting key pluripotency genes (e.g., Nanog, Sox2, Tet1) from progressive epigenetic repression [223]. Finally, a recent study, also aiming at identifying ARTD14-dependent MARylation targets, suggested that ARTD14 modulates cancer-directed host immune responses [181]. Using similar clickable NAD^+^ analogs as described above as well as Af1521-based enrichment for MS, this study unambiguously identified Cys as the ADPr amino acid acceptor side of ARTD14. In contrast to these studies in which ARTD14 is described to exert tumor-progressive functions, another recent study proposed that ARTD14 rather possesses a tumor-suppressive function [224]. In more detail, they described a negative feedback mechanism in which HIF-1-induced transcriptional activation of ARTD14 resulted in the sequestering of HIF-1α and several other oncogenic transcription factors in nuclear bodies where they got degraded by the E3 ubiquitin ligase HUWE1. Of note, all three studies were performed on cells grown in a cell culture. The use of different cell lines and culture conditions could indeed affect the experimental outcomes and thus be at the origin of the inversing results described above. It would therefore be interesting to verify the respective findings in patient-derived samples and to correlate the occurrence of ARTD14-dependent MARylation with cancer progression and prognosis.

In addition to those ARTDs, ARTC1 might also promote cell proliferation and thus potentially favor tumor growth. Early mouse studies, for instance, demonstrated that ARTC1 is related to apoptosis, proliferation and migration of mouse colon CT26 cells [225,226,227], suggesting that ARTC1 is involved in tumor growth, invasion and metastasis. ARTC1 also increased tumor microvessel density in the CT26 model in vivo [228]. Both studies identified the PI3K/Akt pathway to be critical for the observed effects, although the receptor activating this pathway was not identified. Knockdown of ARTC1 stimulated starvation-induced autophagy restrained growth and promoted apoptosis [229]. Furthermore, the proliferation of human umbilical vein’s endothelial cells was increased when cocultured with human epithelial LoVo cells transfected with ARTC1-cDNA, while a shRNA-mediated knockdown of ARTC1 had the opposite effect [228]. In addition, the authors showed that ARTC1 expression correlated with the expression of the vascular endothelial growth factor [228], although the exact molecular mechanism has yet to be fully elucidated. Moreover, through univariate and multivariate analyses, ARTC1 was identified as an independent prognostic factor suggesting that ARTC1 expression is associated with the aggressiveness of glioma [230]. To what extent and how the enzymatic activity of ARTC1 is responsible for the observed effect during tumorigenesis remains elusive.

In contrast to ARTD10, ARTD14 and ARTC1 which are likely to promote tumor growth, ARTD17/PARP6 was reported to be a negative regulator of cell proliferation and forced expression of ARTD17 in HeLa resulted in growth suppression and accumulation of cells in the S-phase [119]. Immunohistochemical analysis revealed that strong ARTD17 staining was often observed in colorectal cancer tissues with well-differentiated histology compared to those with poorly differentiated histology. Furthermore, ARTD17 abundance negatively correlated with the Ki-67 proliferation index and ARTD17-positive colorectal cancer had a better prognosis compared to samples with negative staining.

Although a few targets of the ADP-ribosylation catalyzing sirtuins are known (e.g., Glutamate dehydrogenase (GDH) for SIRT4 [189] or KAP1 for SIRT6 [231]), it remains to be determined if their enzymatic activity also contributes to tumor formation.

### 8.2. A Potential Role of Mono-ARHs in Cancer Progression and Severity

Similar to ARTD17/PARP6, the arginine-specific eraser ARH1 also seems to harbor tumor-protective potential [232]. ARH1-deficient mice showed an increase in tumor extent and frequency in multiple organs, including lung and liver. In line with that, ARH1-deficient and ARH1-mutated mouse embryonic fibroblasts (MEFs) were shown to proliferate faster as compared to their wildtype counterparts and to be more likely to develop tumors when injected into nude mice [233]. In addition to ARH1, the macrodomain-containing ARHs MacroD1 and 2 were also associated with tumor growth and progression [234,235,236,237,238,239]. Both ARHs were described to be mutated or to manifest altered gene expression in the context of different types of cancer. However, while different studies tried to identify the underlying molecular mechanism, the results remain contradictory to a certain extent (reviewed elsewhere [240]), and a clear understanding of how either of the two ARHs integrate with cancer progression and severity remains to be investigated.

### 8.3. Detection of MARylation as a Potential Diagnostic Tool in Cancer Biology

While a potential contribution of ADP-ribosylation and different ARTs in tumorigenesis is well-accepted, the profiling of ADP-ribosylation levels in patient-derived materials has remained challenging due to the lack of suitable detection tools. The above-described improvement of ADP-ribosylation-specific antibodies and antibody-like detection tools has, however, for the first time enabled the characterization of ADP-ribosylation levels in patient-derived samples. In a recent study, the authors made use of different ADP-ribose-binding domain Fc-fusion proteins to detect ARTD1-mediated nuclear ADP-ribosylation in patient-derived materials via Western blotting. Specifically, they found that strong nuclear ADP-ribosylation signals correlated with a better outcome and proposed that this resulted from increased sensitivity towards PARP inhibition [241].

Interestingly, immunohistochemical assessment of cellular ADP-ribosylation levels using a newly developed pan-ADP-ribose-specific antibody revealed that only a minority of the patient-derived samples investigated in this study showed increased nuclear ADP-ribosylation [242]. In strong contrast, cytoplasmic ADP-ribosylation was observed in most tumor types and strong cytoplasmic ADP-ribosylation intensity significantly correlated with a better overall survival in invasive ductal breast cancer, invasive lobular breast cancer and high-grade serous ovarian cancer patients. Intriguingly, the cytoplasmic ADP-ribosylation signal was frequently but not exclusively overlapping with the mitochondrial marker ATP5a, indicating that a major fraction of the observed ADP-ribosylation localized to mitochondria. The strong correlation between the ADP-ribosylation level and cancer severity opens the possibility for ADP-ribosylation detection tools to be used in the future as prognostic markers.

## 9. Future Perspectives

Altogether, the generation of new technologies to detect NAD^+^ ADP-ribose has already significantly pushed our current understanding of the role of ADP-ribosylation far beyond its role in DNA damage. Development and constant improvement of MS-based techniques have enabled the identification of ART-specific ADP-ribosylomes and of ART-specific ADPr amino acid acceptor sites. IF-compatible tools, including antibodies and ADP-ribose-binding-domain Fc-fusion proteins, allowed studying compartment-specific ADP-ribosylation, as well as following the dynamics of the modification over time. In addition, the improvement of NAD^+^ detection tools has enhanced our understanding of how NAD^+^ availability and subcellular distribution influence ART activity and vice versa.

While those improvements are promising, many aspects of ADP-ribosylation require further investigation. Of note, the majority of intercellular ARTs and ADP-ribosylating sirtuins are restricted to catalyze mono-ADP-ribosylation and localize to different cellular compartments. It will be important to next identify their cellular targets as well as their preferred ADP-ribose acceptor sites in a cell culture and in vivo. Comparing homeostatic conditions with ART- and localization-specific stimuli and perturbations will help to elucidate the molecular mechanisms that drive the function of individual ARTs and their targets. Moreover, the synthesis of additional ADP-ribosylated peptides will be essential to generate protein- and modification-specific antibodies to validate ART-specific ADP-ribosylation targets and investigate their involvement in cellular processes. Ultimately, the generation of tissue- and cell type-specific genetically modified mice deficient for the ART of choice would help to gain a better knowledge of how ARTs impact health and diseases.

Due to the lack of suitable tools to analyze cellular ADP-ribosylation in the past, its significance for cancer progression and patient outcome was long elusive. Defining ADP-ribosylation levels of tissue microarrays from different tumors and correlating these with the expression levels of mono-ARTs and ARHs could help to identify new targets and medical indications for future cancer therapies. Given the observed crosstalk between the mitochondria and the nucleus and particularly the activity of ARTD1, a better understanding of mono-ARTs in other compartments might disclose new opportunities for the existing PARP inhibitors in the future.

## Figures and Tables

**Table 1:** Overview on currently available NAD detection tools.

**Chemical Assays**			
**Detected NAD Derivative**	**Type**	**Application and Caveats**	**Reference**
NAD^+^/NADH ratio—indirect	Colorimetric substrate conversion assay, dehydrogenase-based	+ Assessment of NAD^+^/NADH ratios in lysed cells and organs – Correlation-based – Requires lysed material, no dynamic measurements possible	[59,61,62]
**LC- and LC/MS-Based Technologies**			
NAD^+^	Liquid chromatography	+ Assessment of NAD^+^ levels in lysed cells and organs – Requires lysed material, no dynamic measurements possible	[78]
Various NAD^+^ derivatives	Targeted MS	+ Simultaneous assessment of various NAD derivatives in lysed cells and organs – Requires lysed material, no dynamic measurements possible	[79,80,81]
**Genetically Encoded Sensors**			
**Detected NAD Derivative**	**Type**	**Application and Caveats**	**Reference**
NAD^+^—indirect	Organelle-targeted ARTD1/PARP1	+ Measurement of NAD in different subcellular compartments – Indirect and NAD^+^-consuming	[82]
NAD^+^	NAD^+^-binding, fluorescence-based	+ Direct imaging-based NAD^+^ measurements in different subcellular compartments – pH-sensitive	[83]
NAD^+^ and NADPH/NADP^+^	NAD^+^/NADH- and NADP/H-binding, FRET-based	+ Direct imaging- and flow cytometry-based NAD^+^ measurements in different subcellular compartments + pH-sensitive and high dynamic range	[84,85]

**Table 2:** Overview of currently available ADP-ribosylation detection tools.

**Antibodies**			
**Type of ADPR**	**Specificity**	**Potential Application and Caveats**	**Reference**
PAR	PAR	Immunfluorescence (IF), Western blotting (WB) for PAR Unable to detect oligomers and MAR	[161]
Pan-ADP-ribose	MAR, PAR, amino acid-independent	IF, WB for MAR and PAR Compatible with pulldowns and MS Cross-reactivity with nucleotide derivatives and ATP-derived PTMs possible Might have biases towards specific ADPr amino acid acceptor sides	[127,148,155]
MAR	MAR, amino acid-independent	IF, WB for aa- and protein-specific MARylation Potentially compatible with pulldowns and MS	[148]
	MAR attached to Ser or Thr	IF, WB for aa- and protein-specific MARylation Potentially compatible with pulldowns and MS	[148]
**ADP-Ribose-Binding Domains**			
**Type**	**Specificity**	**Potential Application and Caveats**	**Reference**
WWE	PAR, oligo ADPR	IF, WB, chromatin affinity precipitation (ChAP) for PAR Cross-reactivity with nucleic acids possible	[162,163]
Macro(H2A1.1)	PAR, MAR	IF, WB for PAR and MAR Might have a bias towards specific ADPR types	[162]
Macro(Af1521)	PAR, oligo, MAR	IF, WB, ChAP for PAR Compatible with pulldown methods (Immunoprecipitation (IP), MS) Might have a bias towards specific ADPR types.	[162,163,164]
Macro3x (ARTD8/PARP14)	PAR (faint), MAR	IF, WB for PAR Compatible with pulldown methods (IP). Fusion to green fluorescence protein (GFP) allows real-time analyses Might have a bias towards specific ADPR types	[98,162,165]
**Chemical Labeling**			
**Type**	**Specificity**	**Potential Application and Caveats**	**Reference**
Enzymatic labeling of terminal ADP-ribose (ELTA)	PAR, oligo, MAR	Compatible with pulldown methods (IP, MS) Can differentiate between different chain length	[166]
NAD labeling	PAR, MAR	Compatible with pulldown methods (IP, MS)	[167,168,169,170,171]
